# Role of Geriatric Assessment Scores as Predictors of Intensive Therapy Feasibility and Survival in Elderly Patients with Primary CNS Lymphoma

**DOI:** 10.3390/cancers17233759

**Published:** 2025-11-25

**Authors:** Lisa K. Isbell, Annika Vreden, Gabriele Ihorst, Roswitha Uibeleisen, Alexander Friedl, Simone Neumaier, Julia Wendler, Andras Orban, Eliza M. Lauer, Heidi Fricker, Natalie Malenica, Gerald Illerhaus, Elisabeth Schorb

**Affiliations:** 1 Department of Medicine I, Medical Center-University of Freiburg, Faculty of Medicine, University of Freiburg, 79106 Freiburg, Germany; 2 Clinical Trials Unit, Medical Center-University of Freiburg, Faculty of Medicine, University of Freiburg, 79106 Freiburg, Germany; 3 Department of Hematology, Oncology and Palliative Care, Klinikum Stuttgart, 70174 Stuttgart, Germany; 4 Department of Endocrinology, Diabetology and Geriatrics, Klinikum Stuttgart, Prießnitzweg 24, 70374 Bad Cannstatt, Germany

**Keywords:** geriatric assessments, primary central nervous system lymphoma, elderly patients, high-dose chemotherapy, autologous stem cell transplantation

## Abstract

**Background/Objectives:** Short induction followed by high-dose chemotherapy and autologous stem cell transplantation (HCT-ASCT) is effective in newly diagnosed elderly patients with primary central nervous system lymphoma (PCNSL) but associated with significant toxicity. Geriatric assessments (GAs) may help to predict treatment risk and prognosis, yet no standardized GAs exist for PCNSL. Our aim was to evaluate the impact of GA on HCT-ASCT eligibility and survival. **Methods:** We analyzed 65 patients > 65 years treated in the MARiTA and MARTA studies. Treatment comprised 2 cycles of rituximab, HD-MTX and cytarabine followed by HCT-ASCT. GAs at diagnosis were analyzed for progression-free survival (PFS), overall survival (OS) and premature end of treatment (pEOT). **Results:** After median follow-up of 43 months, 12-month PFS/OS were 69.2% (95% CI 56.5–78.9%)/70.8% (58.1–80.2%) from diagnosis and 80.4% (66.6–88.9%)/84.3% (71.1–91.8%) from time of HCT-ASCT. ECOG PS ≥ 2, Lachs geriatric screening (Lachs) ≥30% and Cumulative Illness Rating Scale-Geriatric (CIRS-G) ≥6, ≥7 and ≥8, respectively, were significantly associated with pEOT in univariate analysis (UVA). In multivariate analysis (MVA), CIRS-G remained significant. A composite EBL score (ECOG PS ≥ 2, Barthel Index of Activities of Daily Living (Barthel) < 20, Lachs ≥ 30%) ≤1 predicted successful completion of HCT-ASCT in >90% of patients. ECOG PS ≥ 2 and Barthel < 20 were associated with decreased PFS and OS in UVA; ECOG PS ≥ 2 remained significant in MVA. **Conclusions:** This is the first study to link GA with treatment feasibility in elderly PCNSL patients undergoing intensive therapy. Our results will be validated in the PRIMA-CNS trial (EudraCT 2020-001181-10).

## Simple Summary

Elderly patients with primary central nervous system lymphoma are challenging to treat due to frequent comorbidities, poor performance status and lack of standardized therapy, resulting in worse outcomes compared to younger patients. This study evaluated geriatric assessment scores for their impact on survival outcomes and treatment feasibility in patients undergoing high-dose chemotherapy and autologous stem cell transplantation. Our cohort included 65 patients > 65 years treated within the clinical studies MARiTA and MARTA. A sum score of 3 established geriatric assessment scores, that we named the EBL score, predicted a >90% success rate in completing intensive therapy with improved tumor control. This score could substantially improve clinical decision making regarding optimal treatment strategies for elderly patients with primary central nervous system lymphoma. Therefore, we aim to validate these findings in the ongoing phase III PRIMA-CNS trial.

## 1. Introduction

Primary diffuse large B-cell lymphoma (DLBCL) of the central nervous system (PCNSL) is a highly aggressive non-Hodgkin lymphoma (NHL), exclusively invading the central nervous system compartment. High-dose methotrexate (HD-MTX)-based immunochemotherapy followed by consolidating high-dose chemotherapy and autologous stem cell transplantation (HCT-ASCT) is a widely used treatment approach in patients up to the age of 70 years [1,2,3,4,5]. Although therapeutic approaches have advanced, the overall prognosis of PCNSL patients remains worse than that of systemic NHL patients [6].

Patients older than 60 years account for 50% of all PCNSL cases with rising incidence in the elderly [7,8]. Elderly patients have an inferior prognosis compared to younger patients due to several reasons, (i) lacking treatment standards, (ii) increased treatment-related toxicity, as well as (iii) poor performance status and comorbidities, that require individualized treatment strategies [9,10,11]. The French oculo-cerebral lymphoma network (LOC) nationwide population-based study of 1002 patients diagnosed between 2011 and 2016 showed that whole brain radiotherapy (WBRT) and HCT-ASCT consolidation were administered in only 9% and 2% of cases in patients > 60 years of age [12]. In the analysis of the Texas Cancer Registry from 1995–2017 of 375 PCNSL patients ≥ 65 years, survival did not improve over time despite increasing use of treatment [13]. Intensive treatment approaches including HCT-ASCT are often only offered to patients until 75 years of age [11]. Results of the prospective bicentric MARiTA [14] and subsequent multicentric phase II MARTA study [15] as well as observational and retrospective studies support HCT-ASCT effectiveness in the elderly population [16,17,18].

There is considerable interest within the hemato-oncology community in improving the assessment of elderly cancer patients’ tolerance to treatment, with the goal of individualizing treatment strategies and reducing toxicity. One established approach is the multidimensional geriatric assessment (GA), defined as evaluation of somatic, psychological, functional and social domains, as well as comorbidities, nutrition and frailty [19,20,21]. However, because this approach is time-consuming, in routine clinical practice often only the Eastern Cooperative Oncology Group (ECOG) or Karnofsky performance status (PS) is assessed. Since PCNSL patients often present with a poor ECOG PS of 2–3 due to their untreated lymphoma, assessing pre-morbid PS, meaning the PS before first symptoms of lymphoma occurred, is also important [22]. Comorbidity scores such as the Charlson comorbidity index (CCI), the Cumulative Illness Rating Scale-Geriatric (CIRS-G) or the hematopoietic stem cell index (HCT-CI) have been incorporated into clinical trials and have been found to be associated with outcome and treatment-related mortality in some studies investigating intensive treatment strategies in systemic DLBCL [23,24,25], but there is currently limited data on their significance in PCNSL patients.

The German Cooperative Study Group CNS lymphoma included GAs in the abovementioned MARiTA, MARTA and PRIMA-CNS studies [14,15,26]. In a recently published prospective study, a simplified GA including age, CIRS-G, Barthel and instrumental activity of daily living (IADL) scores for patients with systemic DLBCL has been proposed [24]. To our knowledge, just 2 retrospective studies and 1 study protocol evaluated GA scores in PCNSL patients [27,28,29]. Farhi et al. analyzed CIRS-G, CCI and geriatric (G)8 in a cohort of 35 elderly PCNSL patients that were treated with at least 1 dose of HD-MTX from 2008 to 2015 at 2 French centers. None of the patients received HCT-ASCT. The CIRS-G with cut-off of ≥ 8 was associated with decreased PFS and OS in this cohort. David et al. looked at CIRS-G, presence of any geriatric syndrome and impairment in Barthel in 539 newly diagnosed PCNSL patients ≥ 60 years across 20 US academic centers [28] and showed that CIRS-G ≤ 5 was associated with improved OS. Fourteen percent of these patients received HCT-ASCT consolidation. The G8 score is currently being used to guide treatment in an ongoing phase II, multicenter study in Japan in which PCNSL patients ≥ 70 years receive 3 cycles of rituximab, HD-MTX, procarbazine, vincristine (R-MPV) with or without reduced-dose WBRT and 2 additional courses of high-dose cytarabine (AraC) [29].

In the present study, GA data from 65 patients judged transplant-eligible by the investigators and treated in the MARTA and MARiTA studies were analyzed with regard to premature end of treatment (pEOT) as well as progression-free survival (PFS) and overall survival (OS). To our knowledge, this is the first analysis of the Lachs geriatric screening (Lachs) in elderly lymphoma patients [30].

Our results are currently being validated in the randomized phase III PRIMA-CNS trial (DRKS00024085, EudraCT 2020-001181-10), which compares PFS of elderly, potentially HCT-ASCT-eligible PCNSL patients receiving either R-HD-MTX-AraC induction treatment followed by HCT-ASCT or rituximab, HD-MTX, procarbazine (R-MP) followed by procarbazine maintenance [26].

## 2. Materials and Methods

Sixty-five patients were included in the present study: all 14 patients that were treated in the bicentric MARiTA pilot study from December 2015 until September 2017 as well as the 51 patients from the full analysis set of the phase II multicentric MARTA study from November 2017 until September 2020 [14,15]. These were immunocompetent patients with untreated biopsy-proven PCNSL > 65 years of age, who were transplant-eligible as per the investigator’s judgment. All patients or legal representatives provided written informed consent. The studies conformed to the tenets of the Declaration of Helsinki and were approved by the local ethics committee at the University of Freiburg and the ethics committees of the participating centers. Induction treatment consisted of two 21-day cycles of intravenous HD-MTX 3.5 g/m^2^ (day 1), intravenous AraC 2 g/m^2^ twice daily (days 2 and 3) and intravenous rituximab 375 mg/m^2^ (days 0 and 4) followed by high-dose chemotherapy with intravenous rituximab 375 mg/m^2^ (day 8) only for MARTA patients, intravenous busulfan 3.2 mg/kg (days -7 and -6) and intravenous thiotepa 5 mg/kg (days -5 and -4) plus ASCT. Only patients who achieved (unconfirmed) complete remission, partial remission or stable disease after 2 induction cycles underwent HCT-ASCT.

### 2.1. Data Collection

The following parameters were prospectively collected from MARiTA/MARTA study patients at screening: age, gender, ECOG and Karnofsky PS, CCI, Barthel, MMSE as well as parameters of the international extranodal lymphoma study group (IELSG) score [31] and Memorial Sloan Kettering Cancer Center (MSKCC) score [32]. CIRS-G was prospectively collected only for the MARiTA study patients. In addition, for both study populations the Lachs, presence of a geriatric syndrome as well as CIRS-G for the MARTA patients were assessed retrospectively from the electronic case report forms (eCRFs) as well as patient charts of individual study patients. Presence of any geriatric syndrome was defined as presence of at least 1 of the following conditions: dementia, delirium, depression, osteoporosis, incontinence, falls, failure to thrive or neglect/abuse [28]. For MARiTA study patients, survival data were available until November 2024 (up to 9 years). For the MARTA study patients, survival data were available until March 2023 (up to 6 years).

### 2.2. GA Scores

The Barthel index of ADL, referred to as Barthel, assesses functions that are needed for self-care (feeding, transfer, grooming, toilet use, bathing, walking, stair climbing, dressing and undressing, continence). We used the 0–3 point scoring system for 10 items, adding to a total number of 20 points [33]. The Barthel was assessed prospectively but was missing for 3 patients.

The Lachs geriatric screening, sometimes also referred to as geriatric screening according to Lachs (Lachs), is a screening tool with 15 items including questions about visual and hearing impairment, arm and leg function, mobility, incontinence, nutritional status, cognitive status, depression, social support, hospital stay, falls, polypharmacy and pain and can be carried out in 5–10 min [30,34]. The number of abnormal items defines the score. Since the Lachs was evaluated retrospectively, there was not always complete information available for each item. Therefore, the percentage (abnormal items/number of items where information was available) was used for the analysis.

For both comorbidity scores, the CCI and the CIRS-G, the present lymphoma diagnosis (2 points in the CCI and 4 points in the CIRS-G) was not counted. For the CIRS-G we analyzed 3 cut-offs, ≥6, ≥7, ≥8, respectively, because these were the cut-off points analyzed in the current literature. We chose the cut-off ≥7 for the multivariate logistic regression model because it is an established cut-off in other lymphoma trials [35].

### 2.3. Endpoints

The primary endpoint of this analysis was premature end of treatment (pEOT), defined as patients not reaching the first day of HCT-ASCT due to any reason. Secondary endpoints were PFS calculated from the date of start of treatment until progression or death of any cause, whatever occurred first, and OS calculated from the date of start of treatment until death of any cause. PFS was additionally calculated from the date of ASCT to progression or death of any cause, whatever occurred first, and OS was also calculated from the date of ASCT until death of any cause. Observations were censored at the date the patient was last seen alive/alive without progression, if the event of interest did not occur.

### 2.4. Statistical Analysis

The statistical analyses were conducted with SAS 9.4 (SAS Institute Inc., Cary, NC, USA). All analyses were considered exploratory in nature. The impact of pre-treatment GA scores on the primary endpoint pEOT was analyzed with uni- and multivariate logistic regression models. Results are presented as odds ratios (ORs) with accompanying two-sided 95% confidence intervals (CIs).

PFS and OS rates were estimated and displayed using the Kaplan–Meier method. We explored the impact of pre-treatment GA scores on PFS and OS with Cox proportional hazards models, yielding hazards ratios (HRs) with two-sided 95% CIs. For all 3 endpoints, univariate regression models were assessed first, and based on their results and clinical importance multivariate models were established. Calculated *p*-values (2-sided *p* < 0.05 considered significant) were not considered confirmatory but were used to assess the relevance of prognostic factors in multivariate models. Because of the in-part retrospective design and small sample size (*n* = 65 patients), no power calculations were conducted.

## 3. Results

### 3.1. Patient Characteristics

Sixty-five patients were included in the analysis. Median age was 73 with 19 patients between 75 and 80 years old. Twenty-nine patients initially presented with an ECOG performance status of 2 or more. Table 1 summarizes the patient characteristics at first diagnosis.

### 3.2. Evaluation of Geriatric Assessment Scores Including a Composite ECOG–Barthel–Lachs (EBL) Score with Regard to Premature End of Treatment

**Figure 1:**
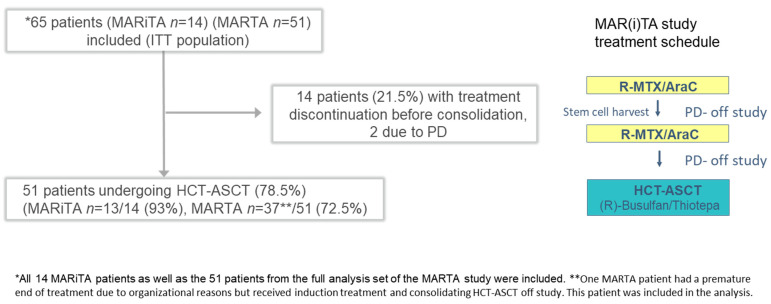
Flow diagram of treatment course and treatment schedule of MAR(i)TA study patients. Abbreviations: HCT-ASCT = high-dose chemotherapy and autologous stem cell transplantation, ITT = intention to treat, R-MTX/AraC = rituximab, MTX, cytarabine, PD = progressive disease.

Of 65 patients that commenced induction treatment, 14 patients did not reach consolidation treatment with HCT-ASCT. Seven patients discontinued treatment due to adverse events during the induction cycle, mainly because of infectious complications. Additionally, 7 patients discontinued treatment before high-dose chemotherapy, due to progressive disease (PD) in 2 patients and due to diverse adverse events or other reasons (gastrointestinal bleeding, cerebrovascular events, patient preference, investigator preference, insufficient stem cell collection) in the other 5 patients (Figure 1).

In univariate logistic regression analysis (UVA) ECOG PS ≥ 2, Lachs ≥ 30% and CIRS-G with the cut-off points ≥6, ≥7 and ≥8, respectively, were significantly associated with pEOT, while Barthel < 20, MMSE < 24, CCI ≥ 2, presence of a geriatric syndrome as well as age ≥ 75 years were not statistically significant (Table 2). In multivariate analysis (MVA) only CIRS-G was significantly associated with pEOT, whereas ECOG PS ≥ 2 almost reached statistical significance (Table 2).

In an attempt to evaluate the established ECOG PS together with extended GA for pEOT, we combined 3 scores with their respective cut-off values into 1 composite ECOG–Barthel–Lachs (EBL) score, assigning 1 point each for ECOG PS ≥ 2, Barthel < 20 (20 meaning full points) and Lachs ≥ 30%.

We decided to include the prospectively assessed Barthel although it did not reach statistical significance in UVA and MVA with regard to pEOT, likely due to missing data in 3 patients and the small sample size, because it is easy to assess and already well established in daily clinical practice.

**Figure 2:**
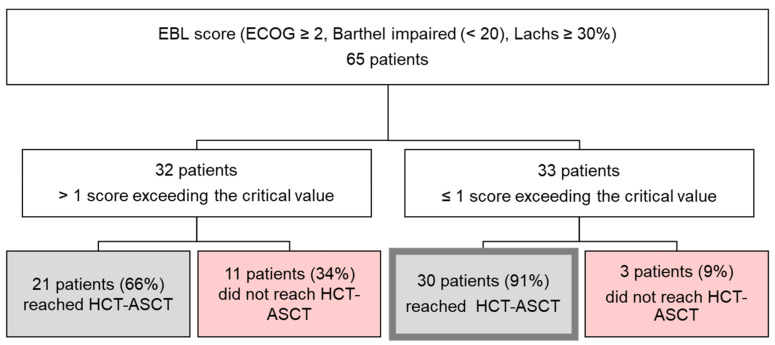
A composite sum score (ECOG–Barthel–Lachs, EBL), including ECOG PS ≥ 2, Barthel Index of ADL < 20 and Lachs ≥ 30% was significantly associated with the risk of pEOT using a cut-off of >1 (*p* = 0.0198; OR = 5.24; 95% CI 1.3–21.09). In contrast, 30/33 (90.9%) patients with an EBL Score of at most 1 reached HCT-ASCT.

Using a cut-off of >1 score points, the EBL score was significantly associated with a higher risk of pEOT with 11/32 (34%) patients not reaching HCT-ASCT. Remarkably, 30/33 (90.9%) patients with an EBL score of at most 1 reached HCT-ASCT (*p* = 0.0198; OR = 5.24; 95% CI 1.3–21.09) (Figure 2).

### 3.3. Survival Outcomes in the MAR(i)TA Cohort

**Figure 3:**
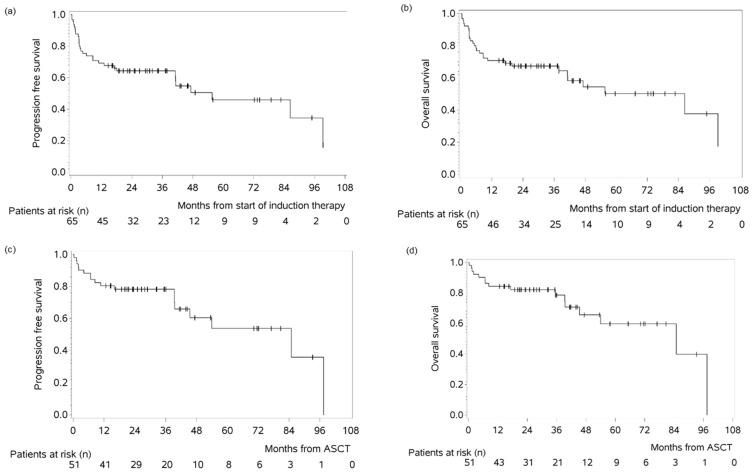
Progression-free survival (PFS) and overall survival (OS) in the MAR(i)TA cohort. PFS (**a**) and OS (**b**) of MAR(i)TA patients from time of start of treatment. PFS (**c**) and OS (**d**) of the 51 MAR(i)TA patients who received consolidation treatment with HCT-ASCT, calculated from date of ASCT.

After a median follow-up of 43 months, PFS and OS at 12 months from time of start of treatment were 69.2% (95% CI 56.2–78.9%) and 70.8% (95% CI 58.1–80.2%), whereas PFS and OS at 12 months from time of HCT-ASCT were 80.4% (95% CI 66.6–88.9%) and 84.3% (95% CI 71.1–91.8%) (Figure 3a–d).

### 3.4. Geriatric Assessment Scores as Prognostic Factors for Survival

**Figure 4:**
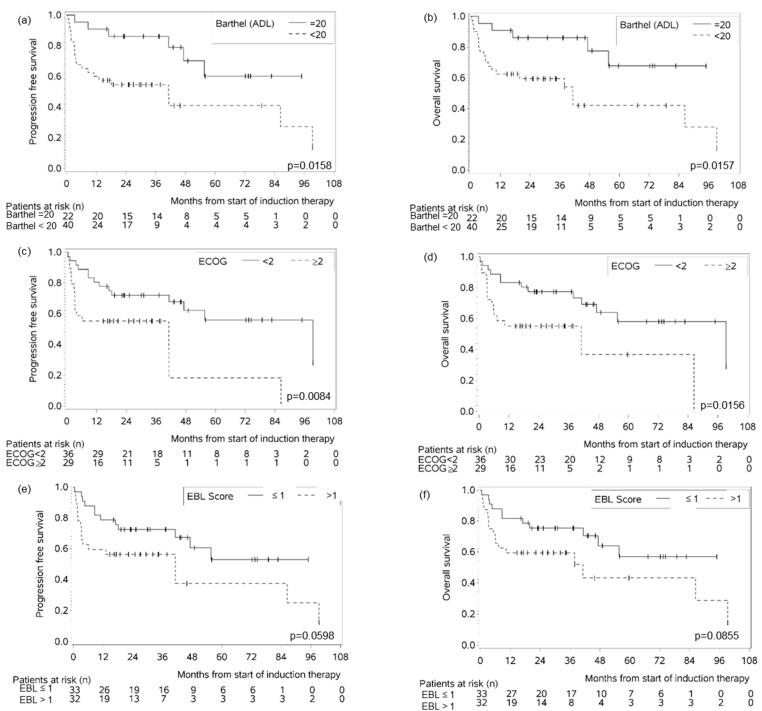
PFS (**a**) and OS (**b**) of MAR(i)TA patients with Barthel < 20 in comparison to =20 as well as PFS (**c**) and OS (**d**) of MAR(i)TA patients with an ECOG ≥ 2 vs. <2. PFS (**e**) and OS (**f**) of MAR(i)TA patients with an EBL score >1 vs. ≤1. The prognostic impact of the EBL score could not be demonstrated at the 5% level.

In UVA Barthel < 20 and ECOG PS ≥ 2 were significantly associated with inferior PFS and OS, whereas for Lachs ≥ 30%, CIRS-G with the cut-off points <6, <7 and <8, respectively, MMSE < 24 points, CCI ≥ 2, geriatric syndrome present and the EBL score did not reach statistical significance at the 5% level (Table 3, Figure 4).

We additionally analyzed the prognostic significance of the EBL score components for PFS and OS in MVA where only an ECOG PS ≥ 2 was found to be significantly associated with inferior PFS and OS (Table 4).

## 4. Discussion

To our knowledge, this is the first study to analyze GA scores in relation to the premature termination of treatment in a cohort of elderly, transplant-eligible PCNSL patients scheduled to undergo intensive systemic treatment, including HCT-ASCT. For this setting, we defined and evaluated a new GA sum score for its potency to determine transplant eligibility, based on the observation that approximately 30% of patients judged as transplant-eligible experience a pEOT, resulting in a worse prognosis. In addition, we analyzed GA scores in relation to survival and provided data on their prognostic impact, enriching the rather scarce literature pool in this field.

Farhi et al. analyzed a cohort of 35 PCNSL patients ≥ 60 years treated with different MTX-based chemotherapy protocols from 2008–2015, none receiving HCT-ASCT, and reported that CIRS-G ≥ 8 was associated with decreased OS and PFS in this cohort in UVA but not MVA and that ECOG had no prognostic impact [27]. In contrast, in our study evaluating HCT-ASCT feasibility in elderly PCNSL patients, ECOG ≥ 2 was significantly associated with decreased PFS and OS in UVA and MVA. Our analysis further revealed that a CIRS-G ≥ 7 was associated with the risk of pEOT in UVA and MVA but interestingly did not have a prognostic impact on PFS or OS.

David et al. retrospectively analyzed CIRS-G, presence of any geriatric syndrome (defined as dementia, delirium, depression, osteoporosis, incontinence, falls, failure to thrive or neglect/abuse) and impairment in Barthel in a cohort of 539 newly diagnosed PCNSL patients ≥ 60 years in the USA and found that all 3 scores significantly affected both PFS and OS in UVA. In MVA older age at diagnosis and worse ECOG PS were associated with inferior PFS and older age, higher CIRS-G score and worse ECOG had an association with inferior OS [28]. Only 14% of these patients received HCT-ASCT consolidation. In our analysis we confirmed the statistically significant prognostic impact of ECOG (in UVA and MVA) and Barthel (in UVA) both on PFS and OS. CIRS-G as well as presence of a geriatric syndrome was not related to survival prognosis in our cohort, possibly due to the lower patient number. To the best of our knowledge our data set is unique as it only contains patients all deemed transplant-eligible by the treating physician and scheduled to undergo HCT-ASCT, in contrast to the other studies evaluating GA in PCNSL in the literature.

We are the first to propose the EBL score that includes ECOG PS, Barthel and Lachs in an attempt to combine clinically relevant, easy to assess scores to help the treating physician in their decision-making process for an individualized treatment strategy. We decided to include the Barthel in our score because it is already used in the daily routine and provides a standardized measure of a patient’s functional status. The EBL score shows a strong positive predictive value for reaching HCT-ASCT (0.91), but its negative predictive value is limited (0.34), as two-thirds of patients with an EBL score > 1 still underwent HCT-ASCT. Including a comorbidity assessment, such as the CIRS-G, CCI or HCT-CI, might help to address this limitation.

The limitations of the study are the limited sample size and missing data for some patients. In addition, despite its prospective study design, some parameters were collected retrospectively and with respective limitations (e.g., Lachs). The EBL score requires prospective validation in a larger patient cohort, including patients for whom transplant eligibility is challenging to assess. Therefore, the EBL score as well as the abovementioned GA scores including comorbidity scores will be evaluated in the ongoing phase III PRIMA-CNS trial where 260 potentially transplant-eligible PCNSL patients > 65 years will be randomized after pre-phase R-HD-MTX to be treated with the MARTA protocol versus R-MP and procarbazine maintenance.

## 5. Conclusions

The prognosis of elderly PCNSL patients has improved, as increasing evidence indicates that intensive treatments such as HCT-ASCT are effective in a subset of patients. To fully exploit this potentially curative approach, patient selection for transplant eligibility, currently based primarily on ECOG PS and clinical judgment, needs to be optimized. Our results, obtained in patients deemed transplant-eligible, suggest that standardized assessment tools beyond ECOG PS may be helpful to more accurately estimate the individual risk associated with intensive therapy in PCNSL patients. The EBL score is a first attempt to incorporate additional, easily assessable parameters to better evaluate patient fitness for HCT-ASCT and to guide potential supportive measures or dose adjustments. However, it needs to be prospectively validated in a larger patient cohort, which is already ongoing in the PRIMA-CNS trial.

## Figures and Tables

**Table 1:** Patient and disease characteristics at first diagnosis.

MAR(i)TA *n* = 65	
Variable	Number (%)
Median age (range), years	73 (65–80)
65–69	19 (29%)
70–74	27 (42%)
75–80	19 (29%)
Sex	
Female	37 (57%)
Male	28 (43%)
ECOG Performance Status	
Grade 0	7 (10.8%)
Grade 1	29 (44.6%)
Grade 2	25 (38.5%)
Grade 3	4 (6.1%)
Serum lactate dehydrogenase	
Elevated	28 (43.1%)
Normal	36 (55.4%)
Not done	1 (1.5%)
Lymphoma in deep brain structures	
Yes	35 (53.8%)
No	26 (40%)
Not done	4 (6.2%)
CSF involvement	
Yes	5 (7.7%)
Suspicious	6 (9.2%)
No	47 (72.3%)
Not done	7 (10.8%)
Ocular involvement	
Yes or suspicious	4 (6.2%)
No	52 (80%)
Not done	9 (13.8%)
Histology	
DLBCL	65 (100%)
IELSG score	
Low 0–1	5 (7.7%)
Intermediate 2–3	34 (52.3%)
High 4–5	12 (18.5%)
Missing	14 (21.5%)
MSKCC score	
class 2	39 (60%)
class 3	26 (40%)

Abbreviations: ECOG = Eastern Cooperative Oncology Group, CSF = cerebrospinal fluid, DLBCL = diffuse large B-cell lymphoma, IELSG = International Extranodal Lymphoma Study Group, MSKCC = Memorial Sloan Kettering Cancer Center.

**Table 2:** Univariate and multivariate analysis of geriatric assessment scores as predictive factors for premature end of treatment.

Univariate Analysis			
Variables	Premature End of Treatment *n* = 14/65		
	OR	95% CI	*p*-Value
CIRS-G ≥6/<6 *n* = 32/33	4.832	1.202–19.431	**0.027**
CIRS-G ≥7/<7 *n* = 25/40	3.937	1.136–13.647	**0.031**
CIRS-G ≥8/<8 *n* = 19/46	4.848	1.387–16.944	**0.013**
ECOG PS ≥2/<2 *n* = 29/36	4.210	1.158–15.311	**0.029**
Barthel <20/=20 *n* = 40/22 missing *n* = 3	3.792	0.758–18.983	0.105
Lachs ≥30%/<30% *n* = 31/34	5.683	1.409–22.929	**0.015**
MMSE <24/≥24 *n* = 23/29 missing *n* = 13	1.065	0.280–4.057	0.927
CCI ≥2/<2 *n* = 18/47	2.438	0.705–8.428	0.159
Geriatric syndrome present *n* = 18/46 missing *n* = 1	0.720	0.173–2.990	0.651
Age ≥75/<75 years *n* = 19/46	0.597	0.146–2.436	0.472
**Multivariate Analysis**			
**Variable**	**Premature end of treatment *n* = 14/65**		
	**OR**	**95% CI**	***p*-Value**
CIRS-G (≥7 vs.<7)	7.295	1.447–36.781	**0.016**
ECOG PS (≥2 vs.<2)	5.794	0.997–33.676	0.050
Barthel (<20 vs. =20)	1.372	0.176–10.709	0.763
Lachs (≥30% vs. <30%)	3.803	0.603–24.004	0.155

*p*-values < 0.05 are shown in bold. Abbreviations: Barthel = Barthel Index of Activities of Daily Living, ECOG PS = Eastern Cooperative Oncology Group Performance Status, CIRS-G = Cumulative Illness Rating Scale-Geriatric, MMSE = Mini-Mental State Examination, CCI = Charlson Comorbidity Index, OR = odds ratio, 95% CI = confidence interval.

**Table 3:** Prognostic factors for survival in univariate analysis.

	Progression-Free Survival			Overall Survival		
Variable	Hazard Ratio	95% CI	*p*-Value	Hazard Ratio	95% CI	*p*-Value
Barthel < 20	2.968	1.179–7.472	**0.0209**	3.203	1.186–8.648	**0.0216**
ECOG PS ≥ 2	2.723	1.257–5.898	**0.0111**	2.563	1.165–5.638	**0.0193**
LACHS ≥ 30%	1.279	0.608–2.688	0.5164	1.249	0.578–2.699	0.5713
CIRS-G < 6	0.717	0.344–1.493	0.3737	0.854	0.401–1.818	0.6824
CIRS-G < 7	0.812	0.389–1.695	0.5784	1.024	0.472–2.224	0.9517
CIRS-G < 8	0.935	0.426–2.051	0.8673	1.025	0.450–2.335	0.9539
MMSE < 24	2.019	0.854–4.771	0.1094	1.925	0.796–4.652	0.1459
CCI ≥ 2	0.897	0.396–2.030	0.7941	0.784	0.331–1.857	0.5796
Geriatric syndrome present	1.464	0.675–3.173	0.3344	1.325	0.590–2.976	0.4951
EBL score (Lachs ≥ 30, Barthel < 20, ECOG ≥ 2) > 1	2.062	0.971–4.379	0.0598	1.983	0.909–4.327	0.0855

*p*-values < 0.05 are shown in bold. Abbreviations: Barthel = Barthel Index of Activities of Daily Living, CCI = Charlson Comorbidity Index, CIRS-G = Cumulative Illness Rating Scale-Geriatric, EBL = ECOG–Barthel–Lachs, ECOG = Eastern Cooperative Oncology Group Performance Status, Lachs = Lachs geriatric screening, MMSE = Mini-Mental State Examination.

**Table 4:** Prognostic factors for survival in multivariate analysis.

Variable	Progression-Free Survival			Overall Survival		
	HR	95% CI	*p*-Value	HR	95% CI	*p*-Value
ECOG PS (≥2 vs.<2)	3.161	1.209–8.269	**0.0190**	3.006	1.121–8.063	**0.0288**
Barthel (<20 vs. =20)	2.222	0.785–6.287	0.1324	2.448	0.825–7.261	0.1066
Lachs (≥30% vs. <30%)	0.636	0.258–1.569	0.3260	0.649	0.257–1.643	0.3617

*p*-values < 0.05 are shown in bold.

## Data Availability

Individual participant data will not be shared. Aggregated participant data from the MARTA study is publicly available on the EudraCT registry, accessed on 24 November 2023 (https://www.clinicaltrialsregister.eu/ctr-search/search?query=2016-001628-72) and as part of the article publication [15], MARiTA study participant data is available in the article publication [14].

## Abbreviations

ADL	Activity of daily living
AraC	cytarabine
CCI	Charlson comorbidity index
CI	Confidence interval
CIRS-G	Cumulative Illness Rating Scale-Geriatric
DLBCL	Diffuse large B-cell lymphoma
EBL	ECOG–Barthel–Lachs
ECOG PS	Eastern Cooperative Oncology Group Performance Status
eCRF	Electronic case report form
GA	Geriatric assessment
G8	Geriatric 8
HCT-ASCT	High-dose chemotherapy and autologous stem cell transplantation
HCT-CI	Hematopoietic cell transplantation–comorbidity index
(HD-)MTX	(High-dose) methotrexate
IADL	Instrumental activity of daily living
IELSG	International Extranodal Lymphoma Study Group
LOC	French Oculo-Cerebral Lymphoma Network
MMSE	Mini-Mental State Examination
MSKCC	Memorial Sloan Kettering Cancer Center
MVA	Multivariate analysis
NHL	Non-Hodgkin lymphoma
OS	Overall survival
PCNSL	Primary central nervous system lymphoma
PD	Progressive disease
pEOT	Premature end of treatment
PFS	Progression-free survival
R	Rituximab
R-MPV	Rituximab, MTX, procarbazine, vincristine
R-MP	Rituximab, MTX, procarbazine
UVA	Univariate analysis
WBRT	Whole brain radiotherapy
